# *N*-(2,5-Dichloro­phen­yl)maleamic acid

**DOI:** 10.1107/S1600536809048715

**Published:** 2009-11-18

**Authors:** K. Shakuntala, B. Thimme Gowda, Miroslav Tokarčík, Jozef Kožíšek

**Affiliations:** a Department of Chemistry, Mangalore University, Mangalagangotri 574 199, Mangalore, India; b Faculty of Chemical and Food Technology, Slovak Technical University, Radlinského 9, SK-812 37 Bratislava, Slovak Republic

## Abstract

The asymmetric unit of the title compound, C_10_H_7_Cl_2_NO_3_, contains two independent mol­ecules. The mol­ecular conformation of each maleamic unit is stabilized by an intra­molecular O—H⋯O_carbon­yl_ hydrogen bond owing to the *anti* disposition of the participating entities. The mean planes through the benzene ring and the amido group are inclined at angles of 45.7 (1) and 40.8 (1)° in the two mol­ecules. In the crystal, the independent mol­ecules self-associate *via* N—H⋯O hydrogen bonds into zigzag ribbons propagating along the *a* axis. The ribbons are weakly coupled by C—H⋯π and C—H⋯O inter­actions.

## Related literature

For related structures, see: Gowda, Foro *et al.* (2009 ▶); Gowda, Tokarčík *et al.* (2009**a* ▶,b* ▶); Leiserowitz (1976 ▶); Lo & Ng (2009 ▶); Prasad *et al.* (2002 ▶).
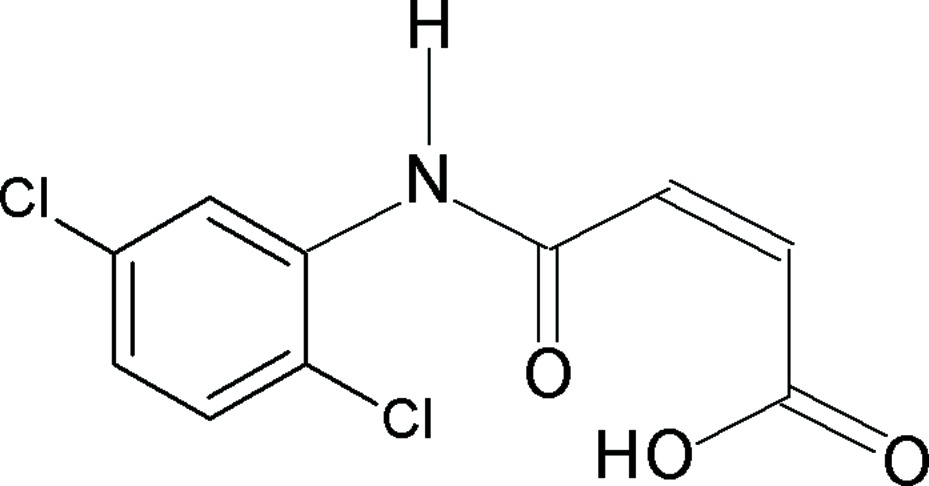


## Experimental

### 

#### Crystal data

C_10_H_7_Cl_2_NO_3_
*M* *_r_* = 260.07
Orthorhombic, 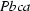

*a* = 13.1618 (2) Å
*b* = 14.6993 (2) Å
*c* = 22.8406 (3) Å
*V* = 4418.95 (11) Å^3^
*Z* = 16
Mo *K*α radiation
μ = 0.58 mm^−1^
*T* = 295 K
0.40 × 0.33 × 0.24 mm

#### Data collection

Oxford Diffraction Xcalibur Ruby Gemini diffractometer
Absorption correction: analytical (*CrysAlis Pro* ; Oxford Diffraction, 2009 ▶) *T*_min_ = 0.836, *T*_max_ = 0.892
61823 measured reflections
4191 independent reflections
3514 reflections with *I* > 2σ(*I*)
*R*_int_ = 0.029

#### Refinement

*R*[*F* ^2^ > 2σ(*F* ^2^)] = 0.028
*wR*(*F* ^2^) = 0.079
*S* = 1.08
4191 reflections
289 parameters
H-atom parameters constrained
Δρ_max_ = 0.22 e Å^−3^
Δρ_min_ = −0.25 e Å^−3^

### 

Data collection: *CrysAlis Pro* (Oxford Diffraction, 2009 ▶); cell refinement: *CrysAlis Pro* ; data reduction: *CrysAlis Pro* ; program(s) used to solve structure: *SHELXS97* (Sheldrick, 2008 ▶); program(s) used to refine structure: *SHELXL97* (Sheldrick, 2008 ▶); molecular graphics: *ORTEP-3* (Farrugia, 1997 ▶) and *DIAMOND* (Brandenburg, 2002 ▶); software used to prepare material for publication: *SHELXL97*, *PLATON* (Spek, 2009 ▶) and *WinGX* (Farrugia, 1999 ▶).

## Supplementary Material

Crystal structure: contains datablocks I, global. DOI: 10.1107/S1600536809048715/tk2578sup1.cif

Structure factors: contains datablocks I. DOI: 10.1107/S1600536809048715/tk2578Isup2.hkl

Additional supplementary materials:  crystallographic information; 3D view; checkCIF report

## Figures and Tables

**Table 1:** Hydrogen-bond geometry (Å, °)

*D*—H⋯*A*	*D*—H	H⋯*A*	*D*⋯*A*	*D*—H⋯*A*
N1—H1*N*⋯O3^i^	0.86	2.07	2.8938 (17)	160
N2—H2*N*⋯O6^ii^	0.86	2.09	2.9263 (17)	164
O2—H2*A*⋯O1	0.82	1.68	2.4979 (15)	175
O5—H5*A*⋯O4	0.82	1.68	2.4846 (15)	166
C7—H7⋯*Cg*2	0.93	2.77	3.6745 (15)	163
C18—H18⋯O5^iii^	0.93	2.58	3.4186 (19)	151

Symmetry codes: (i) 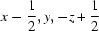; (ii) 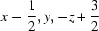; (iii) 
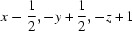
. *Cg*2 is the centroid of the C15–C20 ring.

**Fig. 1.**
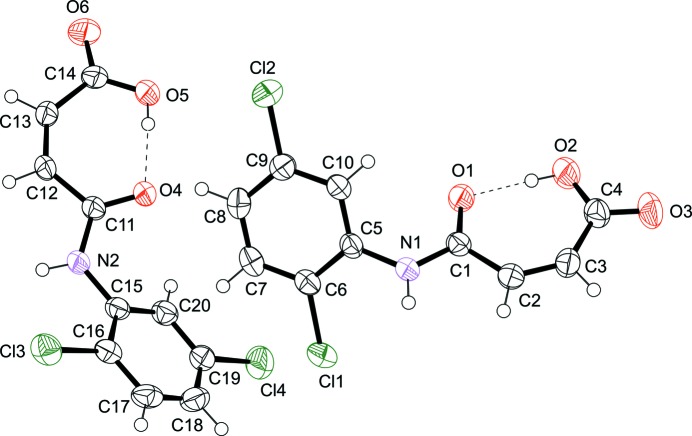
Molecular structure of (I) showing the atom labelling scheme. Displacement ellipsoids are drawn at the 50% probability level. H atoms are represented as small spheres of arbitrary radii.

**Fig. 2.**
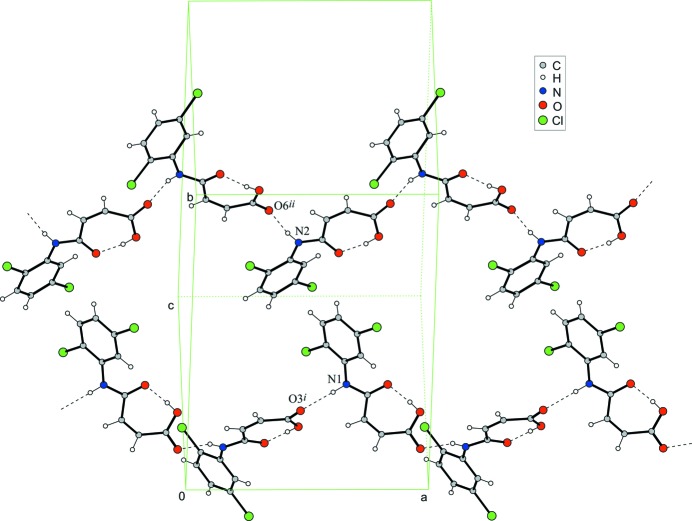
Part of crystal structure of (I) showing 1-D zig-zag supramolecular chains generated by N—H···O hydrogen bonds (dashed lines). Symmetry codes (i) x - 1/2,y,-z + 1/2; (ii) x - 1/2,y,-z + 3/2.

## supplementary crystallographic information

### Comment

As a part of studying the effect of ring- and side-chain substitutions on the crystal structures of biologically significant amides (Gowda, Foro *et al.*, 2009; Gowda, Tokarčík *et al.*, 2009*a,b*; Prasad *et al.*, 2002), the crystal structure of *N*-(2,5-dichlorophenyl)-maleamic acid (I) has been determined. The asymmetric unit of (I) contains two independent molecules (Fig. 1). The conformations of the N—H and C=O bonds in the amide segment of the structure are *anti* to each other, and those of the amide-O atom and the carbonyl-O atom of the acid segment are also *anti* to each other. The *anti* conformation of the C=O and O—H bonds of the acid group is comparatively rare and has been observed previously in *N*-phenylmaleamic acid (Lo & Ng, 2009), *N*-(2,6-dimethylphenyl)maleamic acid (Gowda, Tokarčík *et al.*, 2009*a*), and *N*-(3,4-dimethylphenyl)maleamic acid (Gowda, Tokarčík *et al.*, 2009*b*). The various modes of interlinking carboxylic acids by hydrogen bonds is described elsewhere (Leiserowitz, 1976). Each maleamic moiety includes a short intramolecular hydrogen O–H···O bond (Table 1). The mean planes through the phenyl ring and the amido group –NHCO– form dihedral angles of 45.7 (1) and 40.8 (1) ° in the first and second molecules, respectively. All non-hydrogen atoms of the maleamic moiety in the first molecule fit very well to a plane, having the r.m.s. deviation of fitted atoms 0.013 Å. The mean plane through the maleamic moiety in the second molecule has a r.m.s. deviation of 0.098 Å. In the crystal structure, intermolecular N–H···O hydrogen bonds link self-associated molecules into two distinct zig-zag ribbons propagating in the [1 0 0] direction (Fig. 2). These ribbons are weakly coupled by a C—H···π interaction, with atom C7-H acting as the donor and the aryl ring C15—C20 as the acceptor. The centroid of the C15—C20 ring is denoted *Cg*2 in the Table 1.

### Experimental

A solution of maleic anhydride (0.025 mol) in toluene (25 ml) was treated drop-wise with a solution of 2,5-dichloroaniline (0.025 mol) also in toluene (20 ml) with constant stirring. The resulting mixture was warmed with stirring for 30 min and set aside for an additional 30 min at room temperature for completion of the reaction. The mixture was then treated with dilute hydrochloric acid to remove the unreacted 2,5-dichloroaniline. The resultant solid *N*-(2,5-dichlorophenyl)maleamic acid was filtered under suction and washed thoroughly with water to remove the unreacted maleic anhydride and maleic acid. It was recrystallized to constant melting point from ethanol. Colourless crystals were grown by slow evaporation (room temperature) of an ethanol solution of (I).

### Refinement

H atoms were visible in difference maps and were subsequently treated as riding atoms with distances 0.93Å (CH), 0.86Å (NH) and 0.82Å (OH). The *U*_iso_(H) values were set at 1.2*U*_eq_(C,*N*,*O*).

### Crystal data

**Table:**

C_10_H_7_Cl_2_NO_3_	*F*(000) = 2112
*M**_r_* = 260.07	*D*_x_ = 1.564 Mg m^−^^3^
Orthorhombic, *P**b**c**a*	Mo *K*α radiation, λ = 0.71073 Å
Hall symbol: -P 2ac 2ab	Cell parameters from 31107 reflections
*a* = 13.1618 (2) Å	θ = 1.6–29.5°
*b* = 14.6993 (2) Å	µ = 0.58 mm^−^^1^
*c* = 22.8406 (3) Å	*T* = 295 K
*V* = 4418.95 (11) Å^3^	Block, colourless
*Z* = 16	0.40 × 0.33 × 0.24 mm

### Data collection

**Table:**

Oxford Diffraction Xcalibur Ruby Gemini diffractometer	4191 independent reflections
graphite	3514 reflections with *I* > 2σ(*I*)
Detector resolution: 10.434 pixels mm^-1^	*R*_int_ = 0.029
ω scans	θ_max_ = 25.7°, θ_min_ = 2.3°
Absorption correction: analytical (*CrysAlis PRO*; Oxford Diffraction, 2009)	*h* = −16→16
*T*_min_ = 0.836, *T*_max_ = 0.892	*k* = −17→17
61823 measured reflections	*l* = −27→27

### Refinement

**Table:**

Refinement on *F*^2^	Primary atom site location: structure-invariant direct methods
Least-squares matrix: full	Secondary atom site location: difference Fourier map
*R*[*F*^2^ > 2σ(*F*^2^)] = 0.028	Hydrogen site location: inferred from neighbouring sites
*wR*(*F*^2^) = 0.079	H-atom parameters constrained
*S* = 1.08	*w* = 1/[σ^2^(*F*_o_^2^) + (0.0445*P*)^2^ + 0.705*P*] where *P* = (*F*_o_^2^ + 2*F*_c_^2^)/3
4191 reflections	(Δ/σ)_max_ = 0.001
289 parameters	Δρ_max_ = 0.22 e Å^−^^3^
0 restraints	Δρ_min_ = −0.25 e Å^−^^3^

### Special details

**Table:**

Geometry. All e.s.d.'s (except the e.s.d. in the dihedral angle between two l.s. planes) are estimated using the full covariance matrix. The cell e.s.d.'s are taken into account individually in the estimation of e.s.d.'s in distances, angles and torsion angles; correlations between e.s.d.'s in cell parameters are only used when they are defined by crystal symmetry. An approximate (isotropic) treatment of cell e.s.d.'s is used for estimating e.s.d.'s involving l.s. planes.
Refinement. Refinement of *F*^2^ against ALL reflections. The weighted *R*-factor *wR* and goodness of fit *S* are based on *F*^2^, conventional *R*-factors *R* are based on *F*, with *F* set to zero for negative *F*^2^. The threshold expression of *F*^2^ > σ(*F*^2^) is used only for calculating *R*-factors(gt) *etc*. and is not relevant to the choice of reflections for refinement. *R*-factors based on *F*^2^ are statistically about twice as large as those based on *F*, and *R*- factors based on ALL data will be even larger.

### Fractional atomic coordinates and isotropic or equivalent isotropic displacement parameters (Å^2^)

**Table:**

	*x*	*y*	*z*	*U*_iso_*/*U*_eq_	
C1	0.73471 (10)	−0.00911 (10)	0.32684 (6)	0.0350 (3)	
C2	0.72297 (11)	−0.04413 (10)	0.26641 (7)	0.0415 (3)	
H2	0.6567	−0.0563	0.2547	0.05*	
C3	0.79490 (12)	−0.06048 (11)	0.22658 (7)	0.0441 (4)	
H3	0.7699	−0.0823	0.1912	0.053*	
C4	0.90735 (12)	−0.05043 (11)	0.22796 (7)	0.0441 (4)	
C5	0.63841 (10)	0.02870 (9)	0.41515 (6)	0.0339 (3)	
C6	0.56221 (11)	0.08972 (9)	0.43094 (6)	0.0364 (3)	
C7	0.54753 (12)	0.11305 (10)	0.48908 (7)	0.0415 (3)	
H7	0.4961	0.1535	0.4992	0.05*	
C8	0.60896 (11)	0.07648 (10)	0.53206 (7)	0.0416 (3)	
H8	0.5994	0.0918	0.5712	0.05*	
C9	0.68479 (11)	0.01675 (10)	0.51585 (6)	0.0374 (3)	
C10	0.70068 (11)	−0.00722 (10)	0.45830 (6)	0.0363 (3)	
H10	0.7527	−0.0472	0.4485	0.044*	
N1	0.64725 (9)	0.00060 (8)	0.35603 (5)	0.0376 (3)	
H1N	0.592	−0.0112	0.3374	0.045*	
O1	0.81770 (8)	0.01020 (8)	0.34893 (4)	0.0466 (3)	
O2	0.95290 (9)	−0.01830 (9)	0.27441 (5)	0.0616 (3)	
H2A	0.9105	−0.0062	0.2995	0.092*	
O3	0.95614 (9)	−0.07270 (9)	0.18517 (5)	0.0602 (3)	
Cl1	0.48365 (3)	0.13629 (3)	0.378143 (19)	0.05137 (12)	
Cl2	0.76232 (3)	−0.03099 (3)	0.569340 (18)	0.05382 (13)	
C11	0.54290 (10)	0.29825 (10)	0.65172 (6)	0.0359 (3)	
C12	0.55854 (11)	0.30418 (11)	0.71586 (6)	0.0415 (4)	
H12	0.4998	0.3071	0.7384	0.05*	
C13	0.64578 (11)	0.30582 (11)	0.74530 (6)	0.0420 (3)	
H13	0.6379	0.31	0.7857	0.05*	
C14	0.75294 (11)	0.30223 (11)	0.72582 (7)	0.0410 (3)	
C15	0.41539 (10)	0.31099 (9)	0.57492 (6)	0.0349 (3)	
C16	0.32356 (11)	0.27102 (9)	0.55905 (7)	0.0366 (3)	
C17	0.29294 (12)	0.26877 (10)	0.50117 (7)	0.0444 (4)	
H17	0.2322	0.2405	0.491	0.053*	
C18	0.35224 (13)	0.30829 (11)	0.45862 (7)	0.0471 (4)	
H18	0.3326	0.3062	0.4195	0.057*	
C19	0.44124 (12)	0.35108 (10)	0.47466 (7)	0.0423 (4)	
C20	0.47341 (11)	0.35263 (10)	0.53203 (7)	0.0397 (3)	
H20	0.5339	0.3815	0.5419	0.048*	
N2	0.44678 (9)	0.31040 (9)	0.63412 (5)	0.0386 (3)	
H2N	0.4011	0.3184	0.6606	0.046*	
O4	0.61248 (8)	0.28343 (8)	0.61652 (4)	0.0496 (3)	
O5	0.77523 (8)	0.28321 (10)	0.67163 (5)	0.0590 (3)	
H5A	0.7227	0.2744	0.6532	0.089*	
O6	0.81990 (9)	0.31650 (10)	0.76074 (6)	0.0696 (4)	
Cl3	0.24556 (3)	0.22391 (3)	0.61215 (2)	0.05196 (12)	
Cl4	0.51489 (4)	0.40468 (4)	0.42195 (2)	0.06528 (15)	

### Atomic displacement parameters (Å^2^)

**Table:**

	*U*^11^	*U*^22^	*U*^33^	*U*^12^	*U*^13^	*U*^23^
C1	0.0294 (8)	0.0400 (8)	0.0355 (8)	0.0015 (6)	0.0014 (6)	0.0035 (6)
C2	0.0301 (8)	0.0553 (9)	0.0390 (8)	−0.0023 (6)	−0.0013 (6)	−0.0030 (7)
C3	0.0413 (8)	0.0563 (9)	0.0347 (8)	−0.0033 (7)	0.0042 (7)	−0.0038 (7)
C4	0.0385 (8)	0.0509 (9)	0.0431 (9)	0.0007 (7)	0.0099 (7)	0.0071 (7)
C5	0.0272 (7)	0.0384 (7)	0.0362 (7)	0.0001 (6)	0.0043 (6)	−0.0015 (6)
C6	0.0296 (7)	0.0363 (7)	0.0434 (8)	0.0015 (6)	0.0032 (6)	0.0027 (6)
C7	0.0366 (8)	0.0384 (8)	0.0494 (9)	0.0028 (6)	0.0099 (7)	−0.0059 (7)
C8	0.0443 (8)	0.0428 (8)	0.0376 (8)	−0.0030 (7)	0.0086 (7)	−0.0067 (6)
C9	0.0365 (8)	0.0389 (8)	0.0369 (8)	−0.0043 (6)	0.0000 (6)	0.0017 (6)
C10	0.0303 (7)	0.0398 (8)	0.0390 (8)	0.0046 (6)	0.0033 (6)	−0.0005 (6)
N1	0.0269 (6)	0.0513 (7)	0.0346 (6)	0.0040 (5)	−0.0002 (5)	−0.0021 (5)
O1	0.0311 (6)	0.0708 (7)	0.0381 (6)	−0.0067 (5)	0.0014 (5)	−0.0048 (5)
O2	0.0323 (6)	0.1000 (10)	0.0524 (7)	−0.0044 (6)	0.0077 (5)	−0.0069 (7)
O3	0.0476 (7)	0.0808 (9)	0.0523 (7)	−0.0011 (6)	0.0219 (6)	−0.0027 (6)
Cl1	0.0399 (2)	0.0602 (3)	0.0540 (2)	0.01679 (18)	−0.00180 (18)	0.00427 (19)
Cl2	0.0543 (3)	0.0671 (3)	0.0401 (2)	0.0055 (2)	−0.00553 (18)	0.00738 (18)
C11	0.0255 (7)	0.0469 (8)	0.0352 (8)	−0.0010 (6)	−0.0001 (6)	−0.0043 (6)
C12	0.0253 (7)	0.0661 (10)	0.0331 (8)	0.0018 (7)	0.0046 (6)	−0.0053 (7)
C13	0.0325 (8)	0.0634 (9)	0.0300 (7)	0.0007 (7)	0.0005 (6)	−0.0079 (7)
C14	0.0284 (7)	0.0562 (9)	0.0385 (8)	−0.0011 (6)	−0.0045 (7)	0.0018 (7)
C15	0.0275 (7)	0.0399 (8)	0.0371 (8)	0.0039 (6)	−0.0037 (6)	−0.0061 (6)
C16	0.0295 (7)	0.0344 (7)	0.0460 (8)	0.0021 (6)	−0.0031 (6)	−0.0047 (6)
C17	0.0375 (8)	0.0430 (9)	0.0528 (10)	0.0028 (7)	−0.0152 (7)	−0.0104 (7)
C18	0.0519 (10)	0.0502 (9)	0.0393 (8)	0.0115 (8)	−0.0133 (7)	−0.0074 (7)
C19	0.0418 (9)	0.0452 (8)	0.0398 (8)	0.0119 (7)	0.0017 (7)	0.0013 (7)
C20	0.0309 (8)	0.0447 (8)	0.0435 (9)	0.0003 (6)	−0.0019 (6)	−0.0039 (7)
N2	0.0242 (6)	0.0577 (8)	0.0339 (6)	0.0000 (5)	0.0004 (5)	−0.0058 (5)
O4	0.0305 (6)	0.0850 (8)	0.0334 (5)	0.0094 (5)	0.0014 (5)	−0.0056 (5)
O5	0.0273 (6)	0.1095 (10)	0.0403 (6)	0.0074 (6)	0.0025 (5)	−0.0009 (6)
O6	0.0334 (6)	0.1215 (11)	0.0539 (7)	−0.0072 (7)	−0.0134 (6)	−0.0058 (7)
Cl3	0.0386 (2)	0.0552 (3)	0.0621 (3)	−0.01198 (17)	0.00285 (18)	−0.00156 (19)
Cl4	0.0633 (3)	0.0810 (3)	0.0515 (3)	0.0115 (2)	0.0144 (2)	0.0150 (2)

### Geometric parameters (Å, °)

**Table:**

C1—O1	1.2364 (17)	C11—O4	1.2380 (17)
C1—N1	1.3378 (18)	C11—N2	1.3395 (18)
C1—C2	1.481 (2)	C11—C12	1.482 (2)
C2—C3	1.335 (2)	C12—C13	1.331 (2)
C2—H2	0.93	C12—H12	0.93
C3—C4	1.488 (2)	C13—C14	1.480 (2)
C3—H3	0.93	C13—H13	0.93
C4—O3	1.2143 (19)	C14—O6	1.2070 (19)
C4—O2	1.307 (2)	C14—O5	1.3024 (19)
C5—C10	1.386 (2)	C15—C20	1.385 (2)
C5—C6	1.3931 (19)	C15—C16	1.392 (2)
C5—N1	1.4168 (18)	C15—N2	1.4141 (18)
C6—C7	1.385 (2)	C16—C17	1.382 (2)
C6—Cl1	1.7298 (15)	C16—Cl3	1.7334 (15)
C7—C8	1.381 (2)	C17—C18	1.375 (2)
C7—H7	0.93	C17—H17	0.93
C8—C9	1.380 (2)	C18—C19	1.379 (2)
C8—H8	0.93	C18—H18	0.93
C9—C10	1.377 (2)	C19—C20	1.377 (2)
C9—Cl2	1.7397 (15)	C19—Cl4	1.7349 (16)
C10—H10	0.93	C20—H20	0.93
N1—H1N	0.86	N2—H2N	0.86
O2—H2A	0.82	O5—H5A	0.82
			
O1—C1—N1	122.16 (13)	O4—C11—N2	121.82 (13)
O1—C1—C2	123.52 (13)	O4—C11—C12	123.35 (13)
N1—C1—C2	114.32 (13)	N2—C11—C12	114.83 (12)
C3—C2—C1	128.59 (14)	C13—C12—C11	128.35 (13)
C3—C2—H2	115.7	C13—C12—H12	115.8
C1—C2—H2	115.7	C11—C12—H12	115.8
C2—C3—C4	132.31 (15)	C12—C13—C14	132.04 (14)
C2—C3—H3	113.8	C12—C13—H13	114
C4—C3—H3	113.8	C14—C13—H13	114
O3—C4—O2	120.55 (15)	O6—C14—O5	120.06 (14)
O3—C4—C3	118.83 (15)	O6—C14—C13	119.40 (14)
O2—C4—C3	120.62 (13)	O5—C14—C13	120.54 (13)
C10—C5—C6	119.12 (13)	C20—C15—C16	118.76 (13)
C10—C5—N1	121.19 (12)	C20—C15—N2	121.22 (13)
C6—C5—N1	119.59 (13)	C16—C15—N2	120.01 (13)
C7—C6—C5	120.54 (14)	C17—C16—C15	120.82 (14)
C7—C6—Cl1	119.15 (11)	C17—C16—Cl3	119.13 (12)
C5—C6—Cl1	120.30 (11)	C15—C16—Cl3	120.05 (11)
C8—C7—C6	120.24 (14)	C18—C17—C16	120.01 (14)
C8—C7—H7	119.9	C18—C17—H17	120
C6—C7—H7	119.9	C16—C17—H17	120
C9—C8—C7	118.71 (14)	C17—C18—C19	119.13 (14)
C9—C8—H8	120.6	C17—C18—H18	120.4
C7—C8—H8	120.6	C19—C18—H18	120.4
C10—C9—C8	121.93 (14)	C20—C19—C18	121.43 (15)
C10—C9—Cl2	118.56 (11)	C20—C19—Cl4	118.75 (13)
C8—C9—Cl2	119.50 (11)	C18—C19—Cl4	119.82 (12)
C9—C10—C5	119.44 (13)	C19—C20—C15	119.76 (14)
C9—C10—H10	120.3	C19—C20—H20	120.1
C5—C10—H10	120.3	C15—C20—H20	120.1
C1—N1—C5	125.22 (12)	C11—N2—C15	124.31 (12)
C1—N1—H1N	117.4	C11—N2—H2N	117.8
C5—N1—H1N	117.4	C15—N2—H2N	117.8
C4—O2—H2A	109.5	C14—O5—H5A	109.5
			
O1—C1—C2—C3	−0.1 (3)	O4—C11—C12—C13	8.6 (3)
N1—C1—C2—C3	179.57 (16)	N2—C11—C12—C13	−171.30 (16)
C1—C2—C3—C4	0.2 (3)	C11—C12—C13—C14	−0.2 (3)
C2—C3—C4—O3	178.25 (18)	C12—C13—C14—O6	170.54 (18)
C2—C3—C4—O2	−1.7 (3)	C12—C13—C14—O5	−10.0 (3)
C10—C5—C6—C7	−1.1 (2)	C20—C15—C16—C17	3.2 (2)
N1—C5—C6—C7	175.32 (13)	N2—C15—C16—C17	−178.24 (13)
C10—C5—C6—Cl1	179.64 (11)	C20—C15—C16—Cl3	−176.38 (11)
N1—C5—C6—Cl1	−3.89 (19)	N2—C15—C16—Cl3	2.19 (18)
C5—C6—C7—C8	0.5 (2)	C15—C16—C17—C18	−1.6 (2)
Cl1—C6—C7—C8	179.72 (11)	Cl3—C16—C17—C18	177.97 (11)
C6—C7—C8—C9	0.1 (2)	C16—C17—C18—C19	−1.0 (2)
C7—C8—C9—C10	0.0 (2)	C17—C18—C19—C20	2.0 (2)
C7—C8—C9—Cl2	−179.39 (11)	C17—C18—C19—Cl4	−177.81 (12)
C8—C9—C10—C5	−0.6 (2)	C18—C19—C20—C15	−0.4 (2)
Cl2—C9—C10—C5	178.73 (11)	Cl4—C19—C20—C15	179.43 (11)
C6—C5—C10—C9	1.2 (2)	C16—C15—C20—C19	−2.2 (2)
N1—C5—C10—C9	−175.21 (13)	N2—C15—C20—C19	179.26 (13)
O1—C1—N1—C5	−3.4 (2)	O4—C11—N2—C15	−2.4 (2)
C2—C1—N1—C5	176.88 (13)	C12—C11—N2—C15	177.50 (13)
C10—C5—N1—C1	−45.1 (2)	C20—C15—N2—C11	−39.9 (2)
C6—C5—N1—C1	138.55 (15)	C16—C15—N2—C11	141.55 (14)

### Hydrogen-bond geometry (Å, °)

**Table:**

*D*—H···*A*	*D*—H	H···*A*	*D*···*A*	*D*—H···*A*
N1—H1N···O3^i^	0.86	2.07	2.8938 (17)	160
N2—H2N···O6^ii^	0.86	2.09	2.9263 (17)	164
O2—H2A···O1	0.82	1.68	2.4979 (15)	175
O5—H5A···O4	0.82	1.68	2.4846 (15)	166
C7—H7···Cg2	0.93	2.77	3.6745 (15)	163
C18—H18···O5^iii^	0.93	2.58	3.4186 (19)	151
C20—H20···O4	0.93	2.46	2.8477 (18)	105

Symmetry codes: (i) *x*−1/2, *y*, −*z*+1/2; (ii) *x*−1/2, *y*, −*z*+3/2; (iii) *x*−1/2, −*y*+1/2, −*z*+1.
